# Efficacy of recombinant measles virus expressing highly pathogenic avian influenza virus (HPAIV) antigen against HPAIV infection in monkeys

**DOI:** 10.1038/s41598-017-08326-x

**Published:** 2017-09-20

**Authors:** Tomoko Fujiyuki, Ryo Horie, Misako Yoneda, Takeshi Kuraishi, Fumihiko Yasui, Hyun-jeong Kwon, Keisuke Munekata, Fusako Ikeda, Miho Hoshi, Yuri Kiso, Mio Omi, Hiroki Sato, Hiroshi Kida, Shosaku Hattori, Michinori Kohara, Chieko Kai

**Affiliations:** 1 Laboratory Animal Research Center, The Institute of Medical Science, The University of Tokyo, 4-6-1, Shirokanedai, Minato-ku, Tokyo, 108-8639 Japan; 2 International Research Center for Infectious Diseases, The Institute of Medical Science, The University of Tokyo, 4-6-1, Shirokanedai, Minato-ku, Tokyo, 108-8639 Japan; 3 Amami Laboratory of Injurious Animals, The Institute of Medical Science, The University of Tokyo, 802, Tean Sude, Setouchi-cho, Oshima-gun, Kagoshima, 894-1531 Japan; 4 Department of Microbiology and Cell Biology, Tokyo Metropolitan Institute of Medical Science, 2-1-6, Kamikitazawa, Setagaya-ku, Tokyo, 156-8506 Japan; 5 Research Center for Zoonosis Control, Hokkaido University, North 20, West 10 Kita-ku, Sapporo, Hokkaido, 001-0020 Japan

## Abstract

Highly pathogenic avian influenza virus (HPAIV) is a serious threat not only to domestic fowls but also to humans. Vaccines inducing long-lasting immunity against HPAIV are required. In the present study, we generated recombinant measles virus (MV) expressing the hemagglutinin protein of HPAIV without the multibasic site necessary for its pathogenicity in chickens using the backbone of an MV vaccine strain (rMV-Ed-H5HA) or a wild-type MV-derived mutant (rMV-HL-Vko-H5HA). We examined protective efficacy of the candidate vaccines in the monkey infection model by the challenge with a HPAIV (H5N1). Cynomolgus monkeys inoculated with the candidate vaccines produced both anti-H5 HA and anti-MV antibodies. They recovered earlier from influenza symptoms than unvaccinated monkeys after the challenge with the HPAIV strain. Chest radiography and histopathological analyses confirmed less severe pneumonia in the vaccinated monkeys. Vaccination tended to suppress viral shedding and reduced the interleukin-6 levels in the lungs. Furthermore, the vaccination with rMV-Ed-H5HA of monkeys with pre-existing anti-MV immunity induced the production of anti-H5 HA antibodies. These results suggest that both candidate vaccines effectively reduce disease severity in naïve hosts, and that rMV-Ed-H5HA is a particularly good candidate vaccine against HPAIV infection.

## Introduction

Highly pathogenic avian influenza virus (HPAIV) has continued to threaten human health since H5N1 infection was first recognized in humans in 1997^1^. Although not as prevalent as seasonal influenza, it is considered a serious pathogen because its mortality rate in humans is high^2^. Recent studies have also demonstrated that several mutations in HPAIV confer transmissibility among mammalian hosts^3,4^. Therefore, the development of an effective vaccine against HPAIV is urgently required.

Various types of vaccines for HPAIV are being developed in several countries, including inactivated vaccines, live attenuated vaccines, and DNA vaccines^5^. Inactivated vaccines do not induce long-lasting immunity, and require frequent administration. Therefore, attenuated live vaccines are preferable, based on the strength and duration of their immunostimulation. However, vaccines derived from attenuated HPAIV entail safety issues with the potential occurrence of revertant and reassortant viruses^6^.

Utilization of the measles virus (MV) vector has been proposed to develop live vaccines for infectious diseases^7–10^. Attenuated MV strains had been established for the vaccination of humans, and have excellent characteristics as vectors: (1) the safety of MV vaccines is well known from the history of vaccination; (2) MV vaccines induce cellular immunity and long lasting immunity; and (3) the reverse genetics of MV have been established, and allow foreign genes encoding the antigens of other pathogens to be inserted into the MV genome. We have previously developed several types of recombinant MV (rMV) expressing foreign antigen^11,12^. For example, we used this technique to develop a candidate vaccine against Nipah virus infection and showed that vaccination with the recombinant MV completely protected African green monkeys from a Nipah virus challenge. Therefore, we expect that bivalent vaccine based on MV vaccine vector is useful to develop new vaccines against various emerging infectious diseases.

In this study, we developed a candidate HPAIV vaccine based on the MV vector. We generated rMV expressing an HPAIV antigen and evaluated its efficacy on the challenge with a wild HPAIV (H5N1) strain in a previously established model of HPAIV infection in non-human primate^13^.

## Results

### Generation of recombinant MV expressing H5 hemagglutinin (HA)

**Figure 1:**
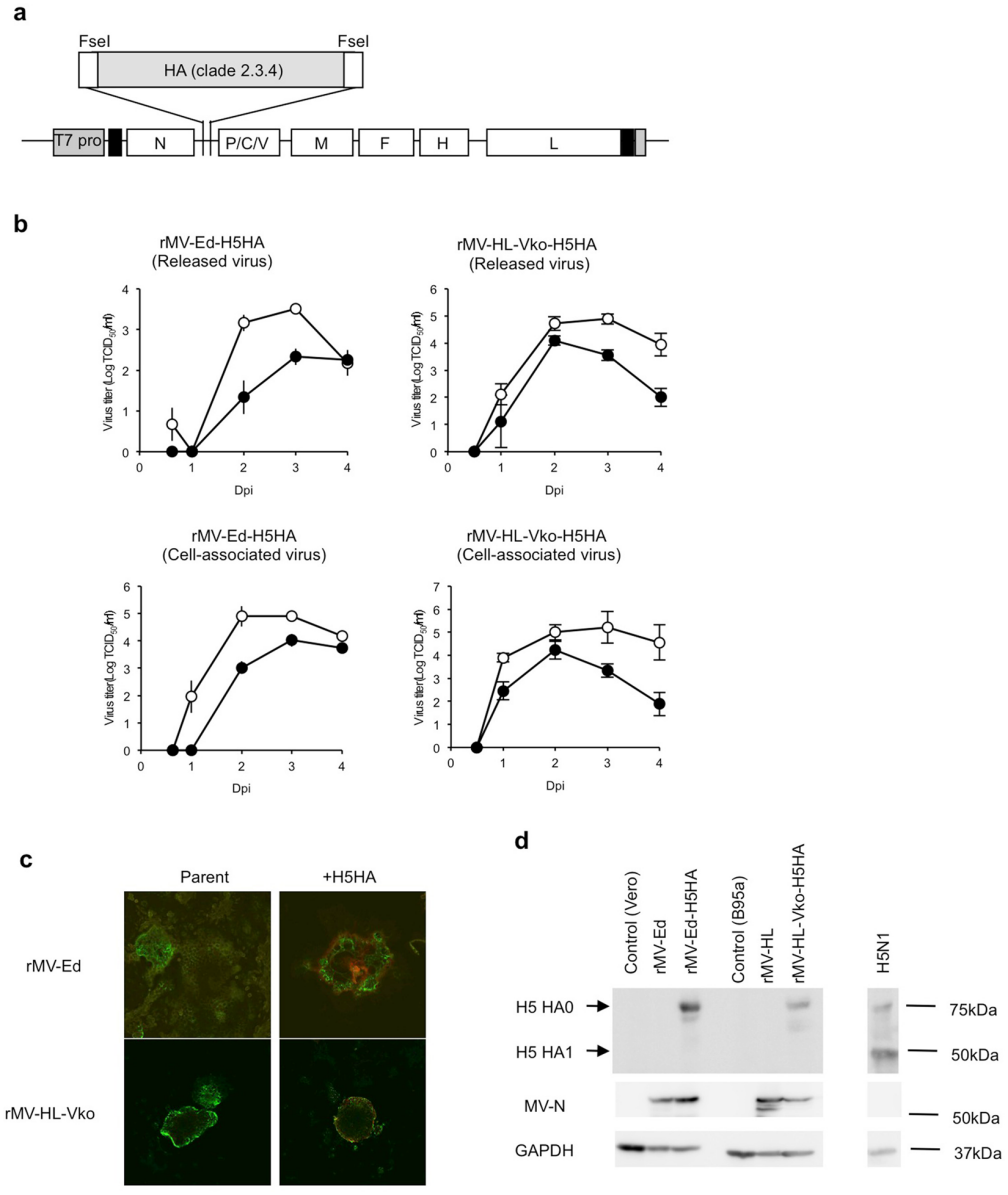
Generation of rMV-H5HA. (**a**) Structure of the full-genome cDNA of rMV-H5HA. (**b**) Comparison of growth curves for rMV-H5HA and the parental rMVs in the corresponding cell lines. Data are averages ± SEM (n = 3). Closed or open dots indicate rMV-H5HA or the parental virus, respectively. Expression of H5 HA protein in cells infected with rMV-H5HA was examined with immunofluorescent microscopy (**c**) and western blotting (**d**) Full-length blots of each protein are shown in Supplemental Figure S1) (c) H5 HA was detected with anti-H5 HA antibody (rabbit) and anti-rabbit IgG antibody conjugated with Alexa Fluor 568, and MV N was detected with an anti-CDV N monoclonal antibody (mouse) and anti-mouse IgG antibody conjugated with Alexa Fluor 488, respectively.

To generate rMV expressing H5 HA, we used a MV vaccine strain (Edmonston) and an attenuated HL strain as the backbone. The attenuated rMV-HL strain was previously generated by genetical modification to induce the deficient expression of the V protein^14^. The H5 HA gene (A/Anhui/1/2005, clade 2.3.4) was synthesized and inserted between the N and P genes of the full-genome cDNA of MV (Fig. 1a). To reduce the possibility that HA will function in cells infected with rMV, which could change the virus’ tropism, the multi basic site of HA necessary for its cleavage was removed. HEK 293 cells were transfected with the resultant plasmid and supporting plasmids and then overlain with B95a cells, and the recombinant viruses (rMV-Ed-H5HA or rMV-HL-Vko-H5HA) were rescued. Each recombinant virus grew in the appropriate permissive cell line (Vero cells for rMV-Ed-H5HA and B95a for rMV-HA-Vko-H5HA), although growth was slower and the maximum titer was lower than those of the corresponding parental viruses (Fig. 1b). An immunofluorescence assay and western blotting analysis demonstrated that H5 HA protein was expressed in the recombinant-virus-infected cells (Fig. 1c,d). Moreover, the expressed HA was not cleaved, unlike that in H5N1-infected cells (Fig. 1d), which is consistent with the design of the candidate vaccines. Therefore, infection with either recombinant virus (rMV-Ed-H5HA or rMV-HL-Vko-H5HA) caused the expression of H5 HA protein *in vitro*. In this paper, rMV-Ed-H5HA and rMV-HL-Vko-H5HA are collectively called ‘rMV-H5HA’.

### Vaccination of cynomolgus monkeys with rMV-H5HA

To examine whether inoculation with rMV-H5HA induces immunity against H5N1 and MV, we inoculated cynomolgus monkeys with rMV-Ed-H5HA or rMV-HL-Vko-H5HA, because these nonhuman primates are susceptive to both MV and H5N1. We measured the antibody titers against MV and H5 with enzyme-linked immunosorbent assays (ELISA) (Table 1). The anti-MV antibody titers increased at 2 weeks post vaccination (wpv) in all three monkeys vaccinated with rMV-Ed-H5HA (#52, #53, and #54). After vaccination with rMV-HL-Vko-H5HA, the anti-MV antibody titers increased at 2 wpv in two of the three monkeys vaccinated (#57 and #58), and at 5 wpv in the third monkey (#56) after boosting. Anti-H5 HA antibodies were induced by rMV-Ed-H5HA in two of the three monkeys (#52 and #53) at 2 wpv, and in the third monkey (#54) at 5 wpv after boosting. Vaccination with rMV-HL-Vko-H5HA also increased the antibody titer against H5 HA (#57 and #58), whereas one monkey needed to be boosted twice (#56). Finally, all the vaccinated monkeys displayed similar antibody titers against MV and H5 HA. Therefore, inoculation with either candidate vaccines induced the production of antibodies against both MV and H5 HA. All the vaccinated monkeys appeared healthy and showed no leukopenia (data not shown), suggesting that H5 HA expression did not affect the safety of rMV.

**Table 1:** Induction of anti-H5 HA and anti-MV antibodies in monkeys after inoculation with rMV-H5HA.

Antigen	Vaccine	Monkey	Weeks post vaccination							
			1	2	3	4	5	6	7	8
MV	Control	#50	0	0	0	0	0	0	0	0
		#51	0	0	0	0	0	0	0	0
		#45	0	0	0	0	0	0	0	0
	rMV-Ed-H5HA	#52	0	400	200	400	1600	1600	3200	3200
		#53	0	800	800	800	6400	6400	12800	3200
		#54	0	800	800	800	12800	6400	6400	3200
	rMV-HL-Vko-H5HA	#56	0	0	0	0	800	800	1600	1600
		#57	0	100	100	800	1600	1600	3200	3200
		#58	0	800	800	800	3200	3200	3200	3200
H5 HA	Control	#50	0	0	0	0	0	0	0	0
		#51	0	0	0	0	0	0	0	0
		#45	0	0	0	0	0	0	0	0
	rMV-Ed-H5HA	#52	0	200	200	100	100	400	200	200
		#53	0	400	200	200	200	400	400	400
		#54	0	0	0	0	200	400	200	200
	rMV-HL-Vko-H5HA	#56	0	0	0	0	0	0	0	400
		#57	0	0	100	200	100	200	200	800
		#58	0	0	100	200	100	100	100	400

Vaccination was performed at 0, 4, and 6 weeks.

### Efficacy of rMV-HA in protecting monkeys from the challenge with a HPAIV (H5N1) strain

**Figure 2:**
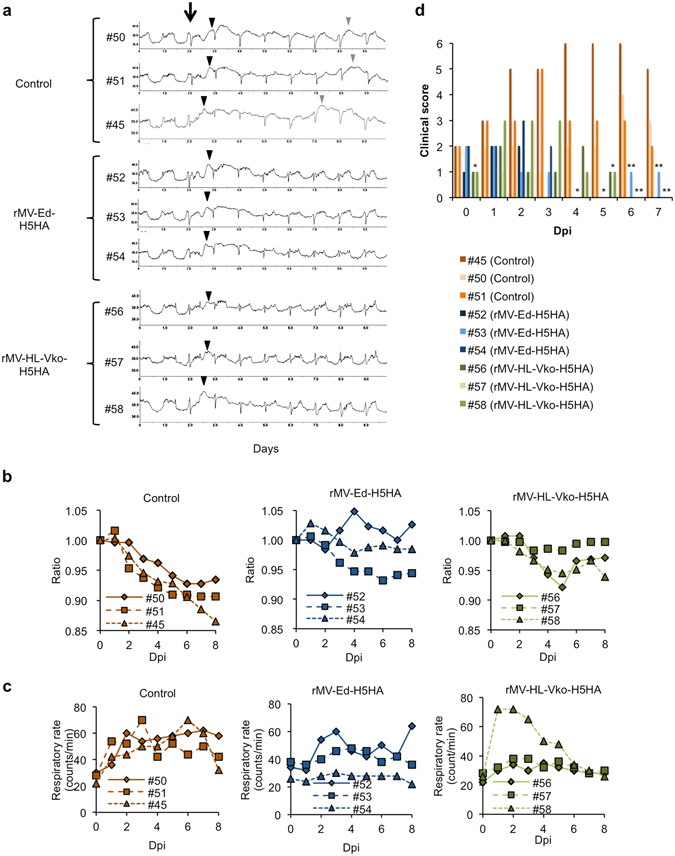
Clinical symptoms in monkeys after HPAIV challenge. (**a**) The vaccinated and unvaccinated monkeys were challenged with HPAIV (arrow). Fever onset was observed on the night of the day of challenge (black arrow heads). A second bout of fever was observed in the unvaccinated monkeys (gray arrow heads). Bodyweight (**b**) and respiratory rate (**c**) were monitored daily after the challenge. (**d**) Clinical symptoms were scored based on Supplemental Table 1. *p < 0.05, **p < 0.01, compared with the unvaccinated control on Dunnett’s test.

To examine the protective effects of rMV-Ed-H5HA or rMV-HL-Vko-H5HA against H5N1 infection, we challenged the vaccinated monkeys with H5N1 (A/whooper swan/Hokkaido/1/2008 clade 2.3.2.1) as previously described^13^. We monitored their body temperature, respiratory rate, heart rate, bodyweight, and appetite. All of the challenged monkeys had fever on the night after the day of challenge (Fig. 2a). Their high temperature persisted during the night, and a second bout of fever occurred in the unvaccinated monkeys (#45, #50, and #51). In contrast, the vaccinated monkeys recovered earlier, their body temperatures showed a circadian rhythm and no second bout of fever occurred (#52, #53, #54, #56, #57, and #58), indicating that fever, especially biphasic fever, was suppressed in the vaccinated monkeys. Tachycardia also persisted longer in the control monkeys than in the vaccinated monkeys (Supplemental Table 1). The unvaccinated monkeys also tended to show a loss of bodyweight, whereas the vaccinated monkeys maintained their bodyweight (Fig. 2b). All the unvaccinated monkeys showed tachypnea, which was more than twice as many as that before the challenge, whereas the vaccinated monkeys, except #58 for rMV-HL-Vko-H5HA, did not (Fig. 2c). When each symptom was scored (Supplemental Table 1), the clinical scores were lower in the vaccinated monkeys than in the unvaccinated monkeys (Fig. 2d). The difference between the control group and the vaccinated groups was significant (one-way repeated-measures ANOVA, p = 0.018), whereas the difference between the monkeys vaccinated with rMV-Ed-H5HA or rMV-HL-Vko-H5HA was not significant. Thus, the clinical symptoms were less severe in the vaccinated monkeys.

**Figure 3:**
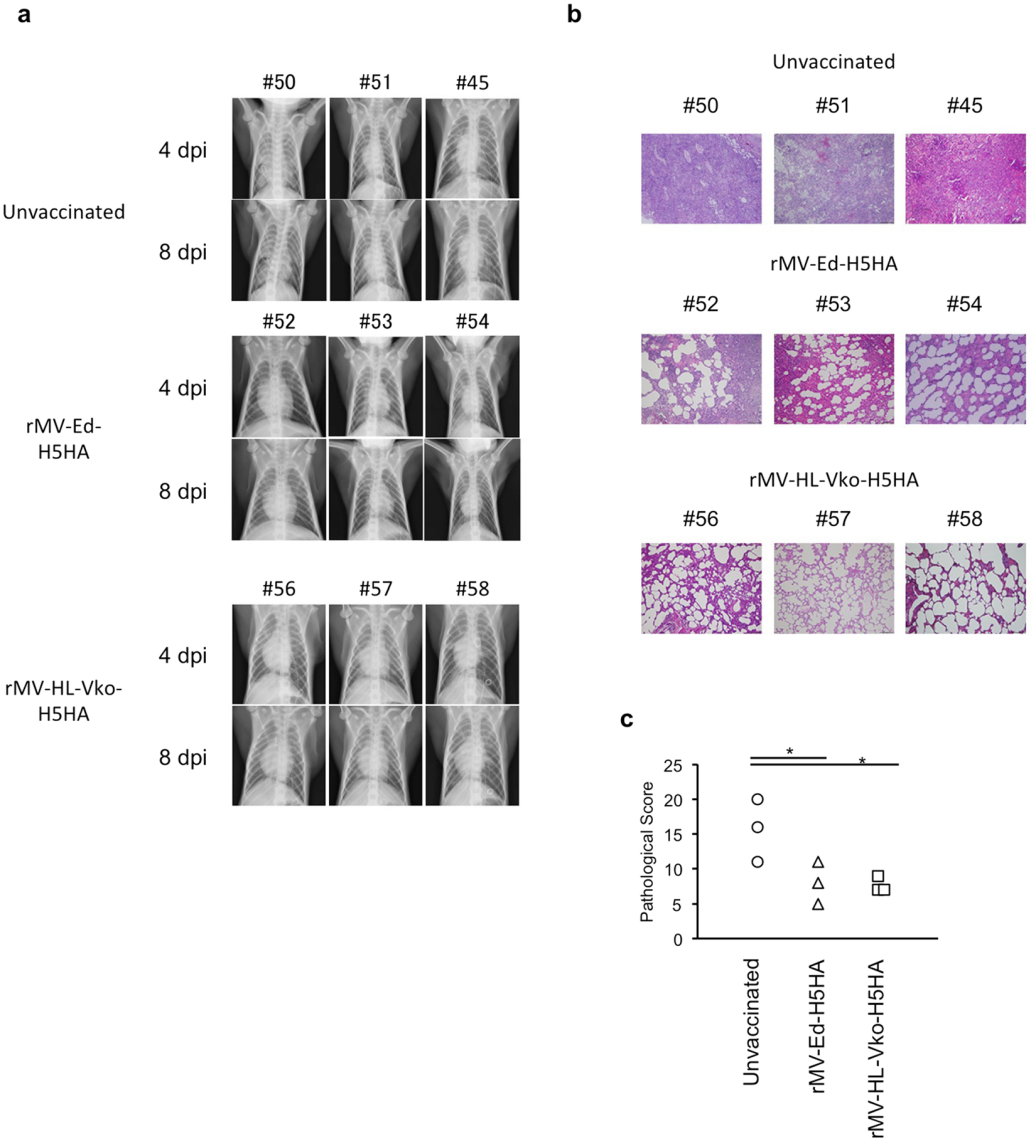
Inflammation of the lungs of HPAIV-challenged monkeys. (**a**) Chest X-rays taken at 4 and 8 dpi are shown. (**b**) Hematoxylin and eosin staining of the lower lobe of the right lung of the monkeys. Original maginifcation, 4× objective lens. (**c**) Histopathology of each lung lobe was scored as: 0, area of pulmonary alveoli was >60% of the observed section; 1, area of pulmonary alveoli was 40–60% of the observed section; 2, area of pulmonary alveoli was 30–40% of the observed section; 3, area of pulmonary alveoli was 20–30% of the observed section; 4, area of pulmonary alveoli was <20% of the observed section. The total score for each individual monkey was used in the analysis. *p < 0.05 compared with the unvaccinated control on Dunnett’s test.

We also examined the progression of pneumonia with chest radiography. In the control monkeys, abnormal density was detected in both the right and left lungs, especially in #50, at 4 days post infection (dpi), and remained until 8 dpi (Fig. 3a). In contrast, among the monkeys vaccinated with rMV-Ed-H5HA, #52 showed slightly abnormal density only in the right lung at 4 dpi; #53 showed abnormal density in both the right and left lungs, but it had decreased at 8 dpi; and #54 showed obvious abnormal density in the right lung, but it had decreased at 8 dpi. In the monkeys vaccinated with rMV-HL-Vko-H5HA, the three monkeys (#56, #57, and #58) showed slightly abnormal density in the right lung, especially in the lower lobes of #57 and #58, at 4 dpi, and it was decreased at 8 dpi. Therefore, the pulmonary density was lower or declined more rapidly in the vaccinated monkeys than in the control monkeys, suggesting that the inflammation in the lungs was less severe in the vaccinated monkeys than in the unvaccinated monkeys.

**Figure 4:**
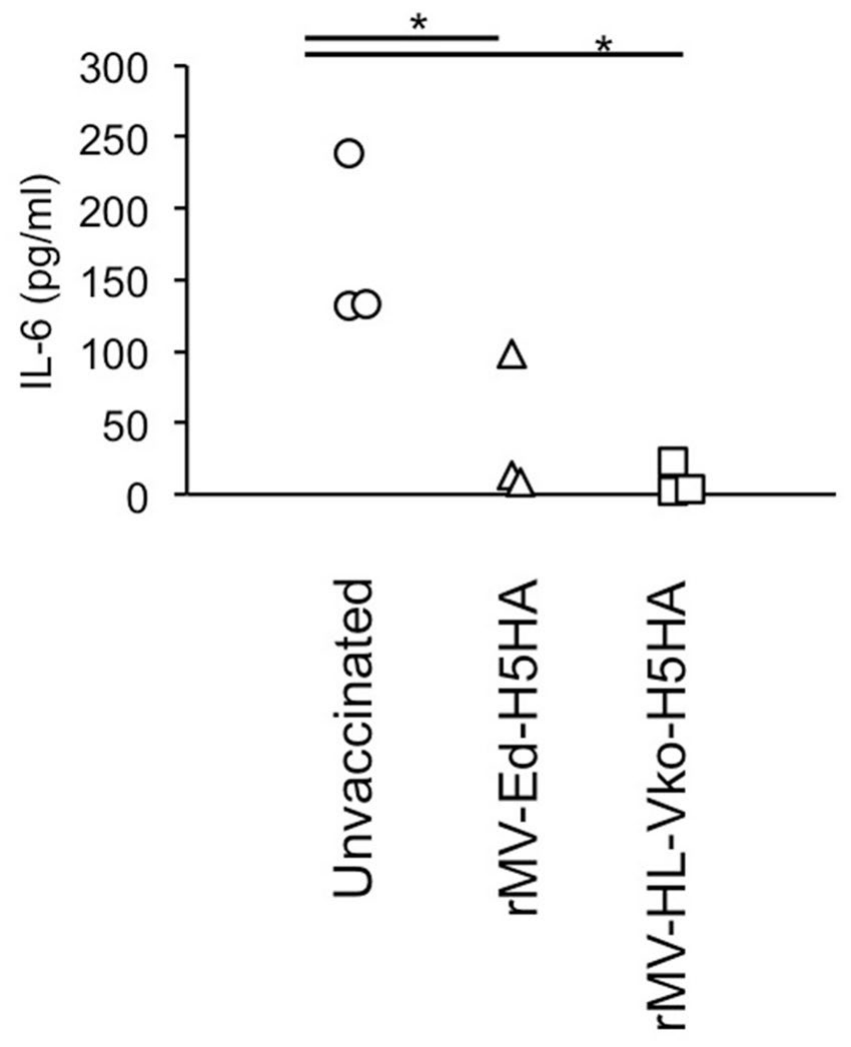
Comparison of IL-6 levels in the lung. IL-6 levels in the lungs of H5N1-challenged monkeys were measured at 8 dpi. Each symbol shows the data from an individual monkey (n = 3 for each group). *p < 0.05 compared with the unvaccinated control on Dunnett’s test.

The monkeys were euthanized at 8 dpi and a histopathological analysis was performed. In the control monkeys, the lung showed severe inflammation (Fig. 3b). There were smaller regions of severe inflammation in the monkeys vaccinated with either candidate vaccine than in the control monkeys (Fig. 3b). To evaluate the severity of inflammation, histopathological scores based on the areas of pulmonary alveoli were compared. Interstitial inflammation was less severe in the vaccinated monkeys than in the unvaccinated monkeys (Fig. 3c). We also compared interleukin-6 (IL-6) levels in the lungs of the control and vaccinated monkeys, because IL-6 levels are known to correlate with influenza severity^15,16^. The levels of IL-6 were lower in the vaccinated group than in the unvaccinated group (Fig. 4).

To further evaluate the effect of vaccination on the progress of HPAIV infection, viral shedding was examined. A reverse transcription (RT)-PCR analysis was used to detect viral RNA in nasal swabs. Viral RNA was detected after several days in the control monkeys, but none was detected or was detected only in one day in the vaccinated monkeys (Table 2), suggesting that vaccination with rMV-H5HA suppressed viral shedding after H5N1 challenge.

**Table 2:** Detection of HPAIV RNA in nasal swabs after challenge.

Vaccine	Monkey	Days post infection							
		1	2	3	4	5	6	7	8
Control	#50	+	+	+	+	−	−	+	−
	#51	−	+	+	−	+	−	−	−
	#45	+	+	−	−	−	−	−	−
rMV-Ed-H5HA	#52	−	−	−	−	−	−	−	−
	#53	+	−	−	−	−	−	−	−
	#54	+	−	−	−	−	−	−	−
rMV-HL-Vko-H5HA	#56	+	−	−	−	−	−	−	−
	#57	+	−	−	−	−	−	−	−
	#58	−	−	+	−	−	−	−	−

RT-PCR was performed. Presence (+) or absence (−) of HPAIV RNA is indicated.

**Figure 5:**
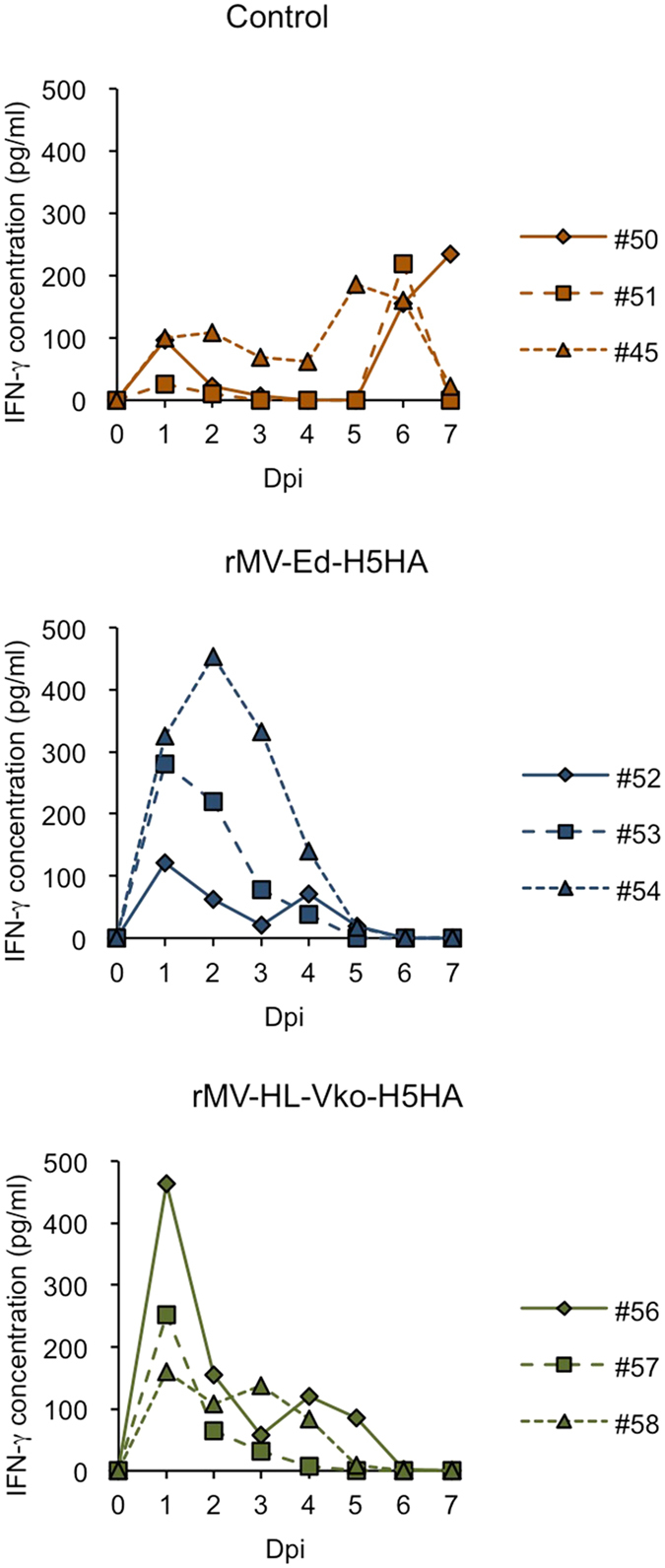
Comparison of IFN-γ levels in the serum or plasma. Serum or plasma samples were collected at 0 dpi (pre-challenge) and 1–7 dpi. IFN-γ levels for individual monkeys are indicated independently.

It is known that MV induces strong cellular immunity. To examine whether the vaccination with rMV-H5HA promotes induction of cellular immunity after H5N1 challenge, we examined interferon-gamma (IFN-γ) levels as an indicator of cellular immune response (Fig. 5). In control monkeys, IFN-γ increased in a biphasic pattern, in a lower level at around 1 dpi and then in a higher level at around 6 dpi. In the monkeys vaccinated with either rMV-Ed-H5HA or rMV-HL-Vko-H5HA, the maximum peak position shifted to 1–2 dpi. This suggests that the vaccination of rMV-H5HA promoted cellular immunity.

Together, these results suggest that vaccination with rMV-Ed-H5HA or rMV-HL-Vko-H5HA reduced the symptoms of influenza after H5N1 infection.

### Influence of pre-existing immunity against MV on vaccination with rMV-H5HA

People are commonly vaccinated with an MV vaccine in childhood, particularly since the initiation of a global MV eradication campaign by the World Health Organization. To examine whether the presence of neutralizing antibodies inhibits the efficacy of the rMV-H5HA vaccines, we vaccinated two monkeys that had previously been vaccinated with an MV vaccine strain with rMV-Ed-H5HA (Table 3). The anti-MV antibody titers were similar in the two monkeys before vaccination with rMV-H5HA (Table 3; 0 wpv). The anti-MV antibody titers clearly increased after one dose rMV-Ed-H5HA (#46 and #47). No anti-H5 HA antibodies were detected in any monkeys after one dose, but the titer increased after the second dose. Therefore, repeated vaccination with rMV-Ed-H5HA induced the production of anti-H5 HA antibodies, even when the host animal displayed pre-existing immunity to MV.

**Table 3:** Antibody production after vaccination with rMV-Ed-H5HA in monkeys possessing anti-MV antibodies.

Antigen	Monkey	Weeks post vaccination							
		0	1	2	3	4	5	6	7
MV	#46	3200	12800	12800	6400	6400	6400	6400	12800
	#47	1600	12800	12800	12800	6400	12800	12800	12800
H5 HA	#46	0	0	0	0	0	800	200	800
	#47	0	0	0	0	0	400	200	800

Vaccination was performed at 0, 4, and 6 weeks.

## Discussion

In this study, we generated recombinant MVs expressing the H5 HA of a HPAIV strain by inserting H5 HA gene between the N and P genes of a vaccine strain or a wild-type-derived attenuated strain of MV. Both candidate vaccines conferred protection against HPAIV challenge in non-human primates.

In our previous studies in which we generated rMV expressing a foreign antigen, the foreign gene was inserted between the N and P genes, the rMV was successfully rescued, and the foreign protein was expressed after infection^11,12^. In this study, rMV-H5HA was also successfully rescued, although the growth of rMV-Ed-H5HA and rMV-HL-Vko-H5HA was slower than that of the parental viruses. Vaccination with rMV-Ed-H5HA or rMV-HL-Vko-H5HA induced the production of anti-H5HA antibodies in cynomolgus monkeys, suggesting that infection with either rMV-H5HA induced an immune response against MV and H5 HA. Vaccination also effectively protected the host animals from severe influenza after H5N1 infection.

When we compared the two candidate vaccines, the protection efficacy based on clinical scores did not differ between rMV-Ed-H5HA and rMV-HL-Vko-H5HA. Therefore, both rMV-Ed-H5HA and rMV-HL-Vko-H5HA are candidate vaccines against HPAIV infection in naïve hosts. Pre-existing immunity against MV must be considered, because most people possess neutralizing antibodies against MV owing to either natural infection or vaccination. Therefore, we examined the effects of prevaccination with MV and demonstrated that rMV-Ed-H5HA induced antibody production against H5 HA even when the monkeys possess MV-neutralizing antibodies, although antibody production was delayed compared with that in MV-naïve monkeys. This suggests that rMV-Ed-H5HA is even effective in the presence of MV-neutralizing antibodies. Similarly, pre-existing immunity against parainfluenza virus 5 (PIV5) did not inhibit the antibody production induced by a bivalent PVI5-based vaccine^17^.

H5 HA used as a vaccine antigen in this study belongs to clade 2.3.4 and the challenge virus belongs to clade 2.3.2.1, thus rMV-H5HA showed heterologous protection. Antigenic variation of HA often affect vaccine efficacy. However, ELISA titers obtained by using recombinant H5 HA of the vaccine antigen and the challenge virus were comparable (200, 400, and 200 for #52, #53, and #54 at 8 wpv). In addition, Yasui *et al*. previously demonstrated that vaccination of a recombinant vaccinia virus expressing the same H5 HA protein as this study induced production of neutralizing antibodies in one of three monkeys tested against the same challenge virus strain (A/whooper swan/Hokkaido/1/2008), and challenge induced the neutralizing antibodies in all of the three monkeys^18^. Also in our case, production of antibodies against H5 HA was boosted by the challenge (Supplemental Table 2), further supporting that immunity induced by vaccination with rMV-H5HA reacts to the challenge virus strain belonging to the different clade.

As for cross-clade protection, Shi *et al*.^19^ reported that antibodies against H5 HA (clade 2.3.4) protect chickens from the challenge of H5N1 (clade 2.3.2.1). Before that report, whereas Zhou *et al*.^20^ reported that neutralizing antibodies against H5 HA of clade 2.3.4 do not neutralize H5 HA of clade 2.3.2.1. The discrepancy may be due to different strains of H5N1 they used. Therefore, possibility of cross-protection may differ dependently on combination of virus strains for antigen and challenge.

Cellular immunity is also believed to contribute to recovery from influenza, by involving cytotoxic T lymphocytes and natural killer cells^21–23^. In this study, increase of IFN-γ in a biphasic patter was observed in the control monkeys, similarly to another monkey infection model of H5N1^24^, whereas the early induction of IFN-γ was observed in the vaccinated monkeys. This suggests that the vaccination with rMV-H5HA helped to prompt cellular immune response against H5N1 infection. We infer that the cellular immune response contributed to remove the H5N1-infected cells from the vaccinated monkeys, resulting in less-severe symptoms.

Existing influenza vaccines are generally inactivated vaccines, thus the effective dose does not confer long-lasting immunity. In contrast, there are many literatures describing that live MV induces long-lasting immunity^25–27^. In addition, it was reported that measles vaccination was successful in protecting people who had no detectable antibodies at exposure^28^, and thus loss of antibody does not necessarily indicate loss of immunity. Thus, it is considered that cellular immunity induced by live MV vaccine is more important than humoral immunity. It remains to be determined whether the immunity induced against the foreign antigen expressed by MV vector-based vaccine is also maintained for a long time.

So far, Newcastle disease virus and PIV5 have been used as paramyxovirus-based vectors for developing a vaccine against H5N1, but their safety and efficacy have not yet been demonstrated in a non-human primate model^29–31^. The MV vector has the advantage that its safety is well known from its long history of vaccination in humans. We have also shown that rMV-Ed-H5HA induced immunity against H5 HA in the host animals even when they were sero-positive for MV. We anticipate that the MV vector can be generally used to develop vaccines against newly emerging infectious diseases.

## Methods

### Ethics statement

All animal experiments were in accordance with the Regulations for Animal Care and Use of The University of Tokyo and were approved by the Animal Experiment Committee at the Institute of Medical Science at The University of Tokyo (approval number: PA10-54).

### Animals and housing

Thirteen cynomolgus monkeys (*Macaca fascicularis*, 5–6 years old, weighing 2.6–3.8 kg, seronegative for MV and H5N1) were imported from the Philippines. Before importation, the sera of the monkeys were imported and their antibody titers against MV and H5N1 were tested with ELISA, as described below, to select individuals lacking the corresponding antibodies. The imported monkeys were maintained at the Amami Laboratory of Injurious Animals at the Institute of Medical Science, The University of Tokyo, as described previously^13^. Transplant of telemetry (TA10CTA-D70, Data Sciences International) was performed after vaccination, and the body temperatures and heart rates of the monkeys were monitored as described previously^13^. The monkeys were euthanized under anesthesia at the end of the experimental period.

### Cells and viruses

HEK 293 cells and Vero cells were maintained in Dulbecco’s modified Eagle’s medium (DMEM) containing 5% fetal calf serum (FCS). B95a cells were maintained in RPMI-1640 containing 5% FCS. Measles virus Edmonston strain (MV-Ed) was propagated in Vero cells in DMEM supplemented with 2% FCS. Viruses were titrated by assessing the 50% tissue culture infectious dose (TCID_50_) in 96-well plates. A/whooper swan/Hokkaido/1/2008 (H5N1 clade 2.3.2.1), originally isolated from a dead wild water bird in Japan was used as described previously^13,32^. All infectious work with H5N1 was performed in P3 laboratories at the Institute of Medical Science, The University of Tokyo.

### Plasmid construction and recombinant virus rescue

To generate an rMV expressing the H5N1 HA protein, we used replication-competent MV-based vectors: pMV-Ed for the Edmonston B strain and pMV-HL-Vko for a previously generated attenuated strain derived from the HL strain^11,14^. An HA cDNA of HPAIV (A/Anhui/1/2005 [H5N1, clade 2.3.4]) whose multi-basic site was deleted, was artificially synthesized^18^. The cDNA was re-amplified with PCR using the following primers: 5′- TG*A* *CGC GT*A TGG AGA AAA ATA GTG CTT CTT C -3′ (*Mlu*I site in italics) and 5′- GCA *GGC CGG CC*A CCT AAA TGC AAA T -3′ (*Fse*I site in italics). The intergenic region between the N and P junction was amplified from pMV-Ed or pMV-HL-Vko by using the following primers: 5′- ﻿AG*G GCC GGC C*TG CCG AGG ACC AGA ACA A -3′ (*Fse*I site in italics) and 5′- AT*A CGC GT*C ATC TGC TCC AAT CGT GGG AG -3′ for pMV-Ed, and 5′- AT﻿*A CGC GT*﻿C ATT GGC TCC AGT CGT GGG AG -3′ for rMV-HL-Vko(*Mlu*I site in italics). The corresponding PCR products were cloned into pGEM-T Easy (Promega). The plasmid was amplified in *Escherichia coli* DH5α and isolated with the Wizard Plus SV Miniprep DNA purification system (Promega), according to the manufacturer’s protocol. After digestion with *Mlu*I and *Nco*I, the N-P intergenic region was inserted into the plasmid containing H5 HA. The fragment of H5 HA connected to the N-P intergenic region was cloned into the FseI site of pMV-Ed or pMV-HL-Vko, and the resulting clones were used to rescue the recombinant MV expressing the H5 HA protein (rMV-Ed-H5HA or rMV-HL-Vko-H5HA), as describe previously^33^.

### Virus characterization

Growth curve of rMV-Ed-H5HA and rMV-HL-Vko-H5HA were generated by infecting Vero and B95a cells, respectively, with the virus at a multiplicity of infection (moi) of 0.01. The virus was allowed to absorb to the cells for 1 h, and the inocula were removed. After medium was added, the cells were incubated at 37 °C. The cells and the supernatants were harvested at 12, 24, 48, 72, and 96 h post infection, and stored at −80 °C. The cells was frozen and thawed three times. The viral titers were determined by calculating the TCID_50_ values with the standard method.

### Immunofluorescence assay

Vero cells were infected with either rMV-Ed-H5HA or the parental rMV-Ed at an moi of 0.01, and B95a cells were similarly infected either rMV-HL-Vko-H5HA or the parental rMV-HL-Vko. The cells were incubated until cytopathic effect (CPE) appeared, were fixed with 4% paraformaldehyde for >20 min at room temperature, and then permeabilized in 0.5% Triton X-100 in phosphate-buffered saline (PBS). The cells were then stained with rabbit polyclonal anti-H5 HA peptide antiserum or mouse polyclonal anti-canine distemper virus N serum^34^ in 10% Block Ace (DS Pharma Biomedical)/PBS solution, and then with goat anti-mouse IgG antibody conjugated with Alexa Fluor 488 (Invitrogen) or anti-rabbit IgG antibody conjugated with Alexa Fluor 568 (Molecular Probes). An imaging analysis was performed with the Fluoview FV1000 system (Olympus).

### Immunoblotting

Vero cells were infected with either rMV-Ed-H5HA or the parental rMV-Ed strain, B95a cells were infected with either rMV-HL-Vko-H5HA or the parental rMV-HL-Vko, and MDCK cells were infected with HPAIV (A/duck/Hokkaido/vac-3/2007 [H5N1])^35^, at an moi of 0.01. After CPE appeared, the cells were harvested, washed with PBS, and lysed in SDS sample buffer (4.6% SDS, 10% 2-mercaptoethanol, 20% glycerol, and 0.01% bromophenol blue in 125 mM Tris–HCl, pH 6.8). Proteins were separated by 10% SDS-polyacrylamide gel electrophoresis and transferred to a polyvinylidene difluoride membrane (Merck Millipore). After the membrane were blocked with TBS containing 5% skim milk and 0.05% Tween 20, they were incubated with a 1:1000 dilution of rabbit polyclonal anti-H5 HA peptide antibody, rabbit polyclonal anti-MV N antibody^36^, or mouse monoclonal anti-glycelaldehyde 3-phospate dehydrogenase (GAPDH) antibody in Can Get Signal® solution (TOYOBO). The membranes were washed three times with TBS containing 0.05% Tween 20 and then incubated with a 1:2000 dilution of anti-rabbit IgG antibody or a 1:1000 dilution of anti-mouse IgG antibody conjugated with horseradish peroxidase (HRP, Dako). The proteins that bound the antibodies were detected with ECL Plus Western Blotting Detection Reagent (GE Healthcare). The reaction was visualized with the LAS1000 system (Fujifilm).

### Immunization and challenge

Three cynomolgus monkeys were subcutaneously vaccinated with rMV-Ed-H5HA or rMV-HL-Vko-H5HA (2 × 10^4^ TCID_50_/monkey). At 4 and 6 weeks post vaccination (wpv), they were inoculated with the same dose as in the primary vaccination. Serum was collected every week after vaccination. At 13 wpv for rMV-Ed-H5HA or 20 wpv for rMV-HL-Vko-H5HA, the monkeys were challenged with a HPAIV (H5N1) strain as described previously^13^. Briefly, a total of 1 × 10^7^ plaque forming units (PFU) (3.3 × 10^6^ PFU/ml) was inoculated to the monkeys intratracheally (2 ml by droplet exposure), intranasally (0.25 ml per nostril by droplet exposure), and orally (0.5 ml by pipette inoculation).

### Measurement of antibody titers

The titer of the antibodies directed the MV and H5 HA protein in the monkey sera were determined with ELISA. Anti-MV antibodies were measured as described previously^11^. To measure the anti-H5 HA antibodies, recombinant H5 HA protein was produced. The H5 HA gene was ligated to the *E. coli* protein expression vector pET21b (Novagen), from which the recombinant protein was expressed as a fusion protein with a histidine tag. Competent BL21 cells were transformed with the plasmid to express the protein at high levels. A 100 mL culture of the transformed cells was induced to express the H5 HA protein in the mid-log growth phase (OD_600_ = 0.4) by the addition of 1 mM IPTG. After incubation for 2 h, the bacteria were collected, lysed with lysis buffer (50 mM Tris-HCl, 500 mM NaCl,20 mM Imidazol pH7.9), and freezed at −80 °C. Thawed protein solution was added with lysozyme at 50 mg/ml, sonicated for 20 sec for 5 times, and then centrifuged at 10,000 × g for 20 min at 4 °C. The recombinant H5 HA was collected in the insoluble fraction. Thus it was dissolved with 6 M urea. The H5 HA protein containing the N-terminal histidine tag in the supernatant was affinity purified with a Ni-Sepharose 6 Fast Flow resin column (GE Healthcare), according to the manufacturer’s protocol. Ninty-six-well microtiter plates were coated overnight with a 0.4 µg/ml solution of the recombinant H5 HA protein diluted in coating buffer (0.1 M carbonate hydrogen carbonate buffer, pH9.6). The wells were blocked with 200 µl of 2% Block Ace in PBS at room temperature for 3 h, and washed with PBS containing 0.05% Tween 20 (PBS-T). The monkey sera (50 µl) were serially diluted two-fold (1:100 to 1:12800) and added to wells. After incubation for 1 h at 37 °C, the wells were washed and incubated for 1 h at 37 °C with 50 µl of HRP-conjugated goat anti-monkey-IgG antibody (1:1000 dilution; Cappel). After the wells were washed again, 100 µl of peroxidase substrate (KPL) was added to each well and the absorbance at 655 nm was measured 30 min later (Bio-Rad).

### Pathological examination

The animals were observed daily for clinical signs. At 1–8 dpi, their bodyweights and respiratory rates were recorded and chest radiography was performed under anesthesia. Nasal swabs were also collected and suspended for RNA extraction, and RT-PCR was performed as described previously^13^. Blood was collected and hematology performed as described previously^13^. At 8 dpi, the animals were euthanized and their lungs were collected. Part of the lungs was fixed in 10% phosphate-buffered formalin. Paraffin-embedded sections were stained with hematoxylin and eosin. Three fields of view were observed in six lobes from each monkey, and the areas of pulmonary alveoli were calculated with Adobe Photoshop CS6 (Adobe Systems). The severity of inflammation was scored as follows: 0, area of pulmonary alveoli was >60% of the observed section; 1, area of pulmonary alveoli was 40–60% of the observed section; 2, area of pulmonary alveoli was 30–40% of the observed section; 3, area of pulmonary alveoli was 20–30% of the observed section; 4, area of pulmonary alveoli was <20% of the observed section. The score for each lobe of an individual monkey were summed and the total score was used in the analysis.

### Measurement of IL-6 level in the lung

L-15 medium (2.5 volume) was added to frozen lung lobe tissues and a tissue homogenate was prepared. The homogenate was centrifuged at 3300 × g for 1 min and the supernatant was used for the assay. The IL-6 levels were measured with a BD Cytometric Bead Array Non Human Primate Th1/Th2 Cytokine Kit (BD Biosciences), according to the manufacturer’s protocol, and a FACSCalibur flow cytometry (BD Biosciences). The data were analyzed with the FCAP Array software (BD Biosciences). The average IL-6 level in the six lung lobes of an individual monkey was calculated, and the average values were used for further analysis.

### Measurement of IFN-γ level

The sera or plasma collected from individual monkeys were heat inactivated, and then IFN-γ levels were measured with a commercial ELISA kit (Monkey IFN-γ ELISA kit, MABTECH), according to the manufacturer’s protocol.

### Vaccination of monkeys with pre-existing immunity against MV

Two naïve monkeys were inoculated with MV Edmonston strain (2 × 10^4^ TCID_50_/monkey). At 4 and 6 weeks postvaccination, they were inoculated with the same dose as the primary vaccination. Serum was collected several times after vaccination and ELISA were performed. After anti-MV antibodies were produced, the monkeys were vaccinated subcutaneously with rMV-Ed-H5HA subcutaneously.

### Statistical analysis

All statistical analyses were performed with the JMP software (JMP Pro 10.0.2, SAS Institute Inc.).

## Electronic supplementary material

Supplementary Information

## References

[1] Gambotto A, Barratt-Boyes SM, de Jong MD, Neumann G, Kawaoka Y (2008). Human infection with highly pathogenic H5N1 influenza virus. Lancet.

[2] Adams S, Sandrock C (2010). Avian influenza: update. Medical Principles and Practice.

[3] Imai M (2013). Transmission of influenza A/H5N1 viruses in mammals. Virus research.

[4] Linster M (2014). Identification, characterization, and natural selection of mutations driving airborne transmission of A/H5N1 virus. Cell.

[5] El Sahly HM, Keitel WA (2008). Pandemic H5N1 influenza vaccine development: an update. Expert review of vaccines.

[6] Luke CJ, Subbarao K (2006). Vaccines for pandemic influenza. Emerging infectious diseases.

[7] Brandler S, Tangy F (2008). Recombinant vector derived from live attenuated measles virus: potential for flavivirus vaccines. Comparative immunology, microbiology and infectious diseases.

[8] Naim HY (2013). Applications and challenges of multivalent recombinant vaccines. Human vaccines & immunotherapeutics.

[9] Naim HY (2015). Measles virus. Human vaccines & immunotherapeutics.

[10] Sato H, Yoneda M, Honda T, Kai C (2012). Morbillivirus receptors and tropism: multiple pathways for infection. Frontiers in microbiology.

[11] Satoh M (2010). Evaluation of a recombinant measles virus expressing hepatitis C virus envelope proteins by infection of human PBL-NOD/Scid/Jak3null mouse. Comparative immunology, microbiology and infectious diseases.

[12] Yoneda M (2013). Recombinant measles virus vaccine expressing the Nipah virus glycoprotein protects against lethal Nipah virus challenge. PLoS One.

[13] Fujiyuki T (2013). Experimental infection of macaques with a wild water bird-derived highly pathogenic avian influenza virus (H5N1). PLoS One.

[14] Sato H (2008). Measles virus induces cell-type specific changes in gene expression. Virology.

[15] Paquette SG (2012). Interleukin-6 is a potential biomarker for severe pandemic H1N1 influenza A infection. PLoS One.

[16] Skoner DP, Gentile DA, Patel A, Doyle WJ (1999). Evidence for cytokine mediation of disease expression in adults experimentally infected with influenza A virus. The Journal of infectious diseases.

[17] Chen Z (2012). Evaluating a parainfluenza virus 5-based vaccine in a host with pre-existing immunity against parainfluenza virus 5. PLoS One.

[18] Yasui F (2016). Sensitization with vaccinia virus encoding H5N1 hemagglutinin restores immune potential against H5N1 influenza virus. Scientific reports.

[19] Shi S (2016). Cross-clade protective immune responses of NS1-truncated live attenuated H5N1 avian influenza vaccines. Vaccine.

[20] Zhou F (2012). A triclade DNA vaccine designed on the basis of a comprehensive serologic study elicits neutralizing antibody responses against all clades and subclades of highly pathogenic avian influenza H5N1 viruses. Journal of virology.

[21] Grant EJ, Quinones-Parra SM, Clemens EB, Kedzierska K (2016). Human influenza viruses and CD8(+) T cell responses. Curr Opin Virol.

[22] Weiss ID (2010). IFN-gamma treatment at early stages of influenza virus infection protects mice from death in a NK cell-dependent manner. J Interferon Cytokine Res.

[23] Yap KL, Ada GL (1978). The recovery of mice from influenza A virus infection: adoptive transfer of immunity with influenza virus-specific cytotoxic T lymphocytes recognizing a common virion antigen. Scand J Immunol.

[24] Shinya K (2012). Integrated clinical, pathologic, virologic, and transcriptomic analysis of H5N1 influenza virus-induced viral pneumonia in the rhesus macaque. Journal of virology.

[25] Markowitz LE, Preblud SR, Fine PE, Orenstein WA (1990). Duration of live measles vaccine-induced immunity. Pediatr Infect Dis J.

[26] Amanna IJ, Carlson NE, Slifka MK (2007). Duration of humoral immunity to common viral and vaccine antigens. N Engl J Med.

[27] Naniche D (2009). Human immunology of measles virus infection. Curr Top Microbiol Immunol.

[28] Samb B (1995). Serologic status and measles attack rates among vaccinated and unvaccinated children in rural Senegal. Pediatr Infect Dis J.

[29] Cornelissen LA (2012). Protective efficacy of Newcastle disease virus expressing soluble trimeric hemagglutinin against highly pathogenic H5N1 influenza in chickens and mice. PLoS One.

[30] Ge J (2007). Newcastle disease virus-based live attenuated vaccine completely protects chickens and mice from lethal challenge of homologous and heterologous H5N1 avian influenza viruses. Journal of virology.

[31] Li Z (2013). Recombinant parainfluenza virus 5 expressing hemagglutinin of influenza A virus H5N1 protected mice against lethal highly pathogenic avian influenza virus H5N1 challenge. Journal of virology.

[32] Okamatsu M (2010). Antigenic, genetic, and pathogenic characterization of H5N1 highly pathogenic avian influenza viruses isolated from dead whooper swans (*Cygnus cygnus*) found in northern Japan in 2008. Virus Genes.

[33] Terao-Muto Y (2008). Heparin-like glycosaminoglycans prevent the infection of measles virus in SLAM-negative cell lines. Antiviral research.

[34] Masuda M (2006). Characterization of monoclonal antibodies directed against the canine distemper virus nucleocapsid protein. Comparative immunology, microbiology and infectious diseases.

[35] Soda K (2008). Development of vaccine strains of H5 and H7 influenza viruses. The Japanese journal of veterinary research.

[36] Hagiwara K (2008). Phosphorylation of measles virus nucleoprotein upregulates the transcriptional activity of minigenomic RNA. Proteomics.

