# Supplementary material for: Efficacy of recombinant measles virus expressing highly pathogenic avian influenza virus (HPAIV) antigen against HPAIV infection in monkeys
Source: Sci Rep. 2017 Sep 20;7:12017. doi: 10.1038/s41598-017-08326-x (PMC5607339; doi:10.1038/s41598-017-08326-x)

**Supplementary Information**

**Efficacy of recombinant measles virus expressing highly pathogenic avian influenza virus (HPAIV) antigen against HPAIV infection in monkeys**

Tomoko Fujiyuki^1^, Ryo Horie^1,2^, Misako Yoneda^1^, Takeshi Kuraishi^2,3^, Fumihiko Yasui^4^, Hyun-jeong Kwon^1^, Keisuke Munekata^4^, Fusako Ikeda^1^, Miho Hoshi^1^, Yuri Kiso^1^, Mio Omi^1^, Hiroki Sato^1^, Hiroshi Kida^5^, Shosaku Hattori^3^, Michinori Kohara^4^, Chieko Kai^1,2, #^

**Supplemental Table 1. Symptoms observed after challenge with H5N1.**

| Vaccine | Monkey | Dpi | | | | | | | |
| --- | --- | --- | --- | --- | --- | --- | --- | --- | --- |
|  |  | 0 | 1 | 2 | 3 | 4 | 5 | 6 | 7 |
| Control | #45 | F, H | F, H, A | F, H, A, L, T | F, H, A, L, T | F, H, B, A, L, T | F, H, B, A, L, T | F, H, B, A, L, T | H, B, A, L, T |
|  | #50 | F, H | F, H | F, T | F | F, H | H, B | F, H, B, T | H, B, T |
|  | #51 | F, H | F, H, L | F, H, A | F, H, B, A, T | F, H, B | F, H, B | F, H, B | H, B |
| rMV-Ed-  H5HA | #52 | F | F, L | F, L | ND | ND | ND | ND | ND |
|  | #53 | F, H | F, H | L | L | ND | ND | B | B |
|  | #54 | F, H | F, H | F, A, L | F, L | ND | ND | ND | ND |
| rMV-HL-Vko-H5HA | #56 | F | F, H | H | A | B, A | B | ND | ND |
|  | #57 | F | H | H | ND | ND | ND | ND | ND |
|  | #58 | F | H, A, T | H, A, T | H, A, T | H | B | ND | ND |

F; fever, H; hyper heart rate, B; bodyweight loss, A; appetite loss, L; leukopenia, T; tachypnea, ND; no detected symptoms.

**Supplemental Table 2. Induction of anti-H5 HA antibodies after the challenge with H5N1.**

| ID/ Dpi | 0 | 1 | 2 | 3 | 4 | 5 | 6 | 7 | 8 |
| --- | --- | --- | --- | --- | --- | --- | --- | --- | --- |
| #50 | 0 | 0 | 0 | 0 | 0 | 0 | 0 | 0 | 0 |
| #51 | 0 | 0 | 0 | 0 | 0 | 0 | 0 | 0 | 0 |
| #52 | 100 | 200 | 200 | 200 | 200 | 400 | 800 | 6400 | 12800 |
| #53 | 400 | 200 | 200 | 200 | 200 | 800 | 3200 | 12800 | 12800 |
| #54 | 400 | 100 | 100 | 100 | 200 | 400 | 3200 | 12800 | 12800 |

**Supplemental Figure 1. Full-length blots of H5 HA, MV N, and GAPDH.**

*: H5 HA was detected by using the same membrane as that for GAPDH, thus signals for GAPDH were remained.


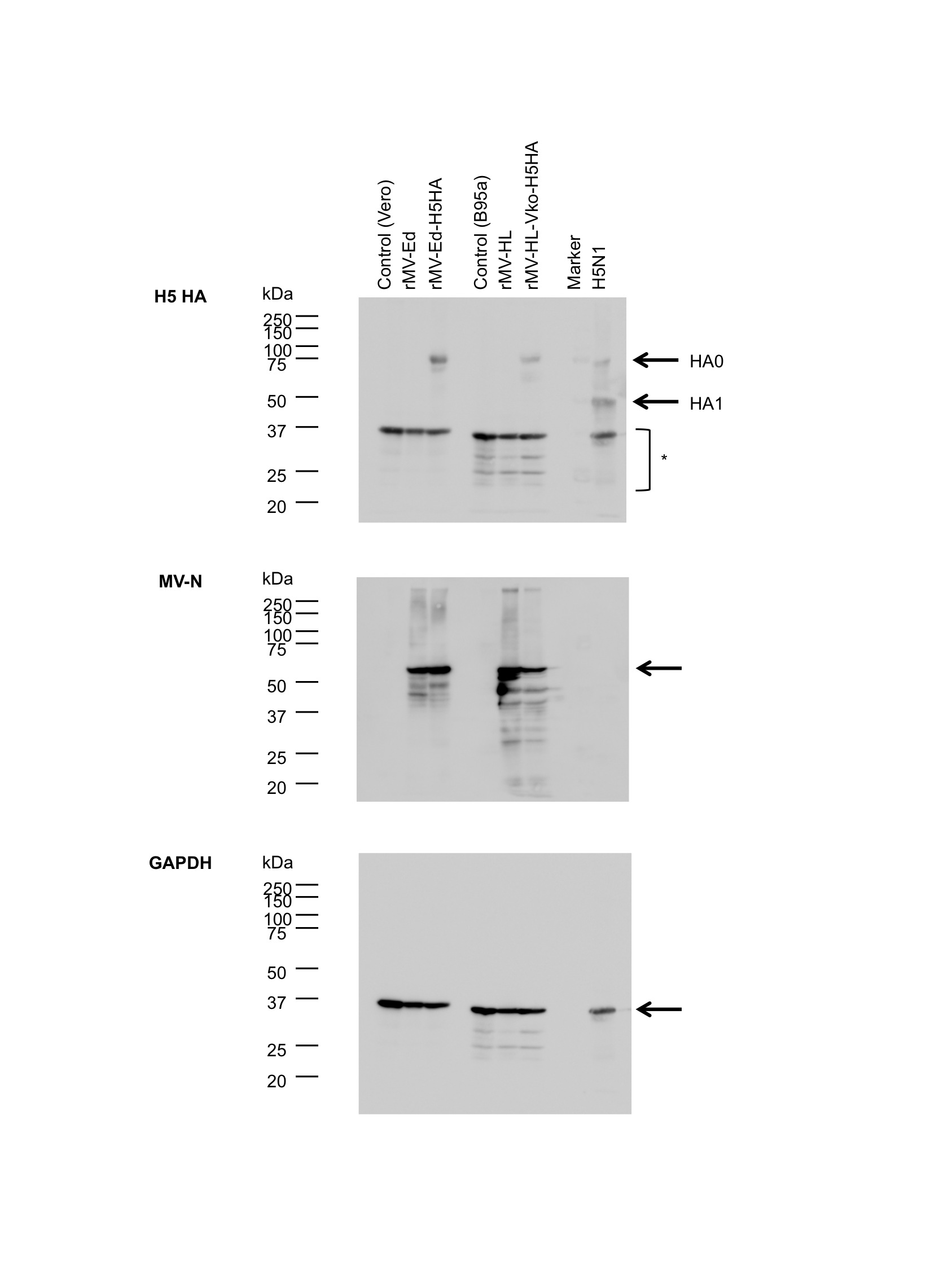

Supplement: Supplementary file 1 — Supplementary Information [file 41598_2017_8326_MOESM1_ESM.doc]
